# Artificial Intelligence Applications for Biomedical Cancer Research: A Review

**DOI:** 10.7759/cureus.48307

**Published:** 2023-11-05

**Authors:** Induni N Weerarathna, Aahash R Kamble, Anurag Luharia

**Affiliations:** 1 Biomedical Sciences, School of Allied Health Sciences, Datta Meghe Institute of Higher Education and Research, Wardha, IND; 2 Artificial Intelligence and Data Science, Datta Meghe Institute of Higher Education and Research, Wardha, IND; 3 Radiotherapy, Jawaharlal Nehru Medical College, Datta Meghe Institute of Higher Education and Research, Wardha, IND

**Keywords:** radiodiagnosis, nanotechnology, personalized treatment, diagnostics, precision medicine, cancer research, artificial intelligence

## Abstract

Artificial intelligence (AI) has rapidly evolved and demonstrated its potential in transforming biomedical cancer research, offering innovative solutions for cancer diagnosis, treatment, and overall patient care. Over the past two decades, AI has played a pivotal role in revolutionizing various facets of cancer clinical research. In this comprehensive review, we delve into the diverse applications of AI across the cancer care continuum, encompassing radiodiagnosis, radiotherapy, chemotherapy, immunotherapy, targeted therapy, surgery, and nanotechnology. AI has revolutionized cancer diagnosis, enabling early detection and precise characterization through advanced image analysis techniques. In radiodiagnosis, AI-driven algorithms enhance the accuracy of medical imaging, making it an invaluable tool for clinicians in the detection and assessment of cancer. AI has also revolutionized radiotherapy, facilitating precise tumor boundary delineation, optimizing treatment planning, and enabling real-time adjustments to improve therapeutic outcomes while minimizing collateral damage to healthy tissues. In chemotherapy, AI models have emerged as powerful tools for predicting patient responses to different treatment regimens, allowing for more personalized and effective strategies. In immunotherapy, AI analyzes genetic and imaging data to select ideal candidates for treatment and predict responses. Targeted therapy has seen great advancements with AI, aiding in the identification of specific molecular targets for tailored treatments. AI plays a vital role in surgery by offering real-time navigation and support, enhancing surgical precision. Moreover, the synergy between AI and nanotechnology promises the development of personalized nanomedicines, offering more efficient and targeted cancer treatments. While challenges related to data quality, interpretability, and ethical considerations persist, the future of AI in cancer research holds tremendous promise for improving patient outcomes through advanced and individualized care.

## Introduction and background

Artificial intelligence (AI) has been widely used in medical specialties [1]. The pioneering work had an impact on a wide range of clinical indications, including ophthalmology, radiology, dermatology, and others. The application of AI technologies to basic biology, pharmacology, and medicine has resulted in numerous performance breakthroughs, with some areas achieving performance comparable to human experts [2]. Early AI approaches were dominated by traditional symbol-based and information-based expert systems. Following that, the advent of machine learning (ML) resulted in revolutionary advances in AI. As a traditional AI technology, ML provides a plethora of algorithms that can improve determination or prediction accuracy with large amounts of high-quality data [3]. Deep learning (DL) is a class of algorithms that use neural network models to solve problems that traditional ML cannot solve [4].

Since the human mind is only capable of digesting small amounts of data quickly, AI has grown exponentially over the past 10 years, indicating its promise as a platform for super-intelligent decision-making [5]. There are thousands of genetic and epigenetic variations in cancer, making it a complex disease. The early detection of genetic mutations and aberrant protein interactions is a promising area for AI-based methods. The safe and moral implementation of AI technology in medical settings is another area of current biomedical research attention [6]. AI-based support for illness risk, diagnosis, prognosis, and therapy prediction could be very helpful to pathologists and doctors. The future of medical advice toward quicker mapping of a novel treatment for each individual is the clinical applications of AI and ML in cancer diagnosis and treatment [7]. Researchers can work together in real time and share knowledge digitally by using an AI-based system method, which has the potential to heal millions of people [5].

Since open-source healthcare statistics are readily available, researchers have created tools to assist in the diagnosis and prognosis of cancer. DL and ML models on distributed datasets offer dependable, fast, and effective answers to issues in cancer therapy [6]. More sophisticated federated learning models can be applied to distributed data analysis. Virtual biopsies, natural language processing (NLP) to infer health trajectories from medical information, and whole-blood cancer diagnosis via deep sequencing are a few of the cutting-edge clinically helpful tools [8]. Oncology mostly uses evidence-based, medication-scoring systems for surveillance monitoring, prognostic staging, diagnosis, treatment, and risk assessment of cancer. These systems have developed from straightforward light-microscopy observations to more complex testing, including gene expression assays and next-generation sequencing of somatic and germ-line genomes [9]. AI has also made it possible to employ medications in concert with one another to treat cancer. Even people with no computer experience can easily engage in AI-powered cancer research when it is backed by robust AI core services and resources. It is anticipated that algorithm-based AI will become more prevalent in digital health care and clinical practices in the future, providing more accurate cancer therapy through data mining, electronic health records (EHR), and radiological image interpretation [10]. By 2021, intelligent AI solutions in the US healthcare sector are expected to save $52 billion, according to marketing cancer research firms. AI intervention in cancer research may be more successful if suitable data for creating ML and DL models are available [11]. The last 20 years have seen an explosion of digital information, which has led to the resurgence of AI and shown promise in all facets of cancer research. Thanks to the quick growth of volume and variety of datasets, a great deal of molecular-level tumor information from cancer patients is now accessible [12].

Though AI is being quickly incorporated into oncologic research, the development of AI solutions is still in its early stages. A small number of AI-based applications, such as those utilized by healthcare facilities and drug manufacturers, have received approval for wider commercial use. Whether AI can take the role of medical doctors as professionals is still up for debate. The development of AI applications for cancer clinical research has been a major topic of discussion in the public discourse regarding AI [13]. As a result, biomedical AI application research has advanced and reached a level of performance that is on par with human experts. Furthermore, AI will give human decision-makers more information, making them an indispensable part of the medical team. Notably, a slew of studies utilizing AI to improve cancer screening/diagnosis and predict treatment outcomes have advanced to clinical trials around the world. Digital pathology, radiology, and genomic data are used in these studies to optimize combination regimen design and determine appropriate chemotherapy and immunotherapy dosing [14].

This review provides an overview of the key concepts of AI in cancer clinical research, including the state of the field over the last two decades, available data, techniques, and current applications in radiation therapy, radiodiagnosis, chemotherapy, surgery, immunotherapy, targeted therapy, and nanotechnology. The difficulties and challenges encountered in translating AI from theoretical studies to real-world clinical use are discussed. We hope that by providing future perspectives, we can help drive meaningful research that will eventually result in AI participation in cancer treatment. Therefore, the aim of this present review is to identify new emerging benefits of AI in healthcare settings.

Artificial intelligence's role in cancer clinical research for the previous two decades

For almost two decades, AI has been employed in the field of cancer research. Promising gains have been made in the field of cancer research, and expert-level performance has been obtained [1]. AI is being used by several businesses and industrial research organizations to identify, diagnose, and treat cancer. IBM (Armonk, NY) is the pioneer in implementing AI in clinical settings, having made a concentrated effort in this direction. To provide medical AI for cancer research, IBM developed Watson in 2014 [15]. Similarly, a group of computer scientists and researchers at Microsoft's research labs are attempting to program biology for cancer treatment using ML and NLP. As a result, AI is poised to have a game-changing impact on improving diagnosis accuracy and speed, assisting treatment option suggestions and recommendations, and ultimately leading to better prognosis outcomes [10].

## Review

Search methodology

To find appropriate content for this review article that looks at AI applications in the field of cancer research, a thorough search method was used. The objective was to give a thorough review of the state of the art, the data at hand, the methods, and the applications in many areas of cancer clinical research. Several scholarly databases, such as PubMed, Web of Science, Scopus, and Google Scholar, were used for information retrieval from 2006 to the present to make sure the most recent study findings were included. These databases were systematically queried using precise keywords related to the subject matter, such as "Artificial Intelligence," "Cancer Research," "Precision Medicine," "Diagnosis," "Treatment," "Radiology," and "Nanotechnology." This well-defined keyword strategy was employed to ensure that studies at the intersection of AI and cancer research were comprehensively captured.

**Figure 1:**
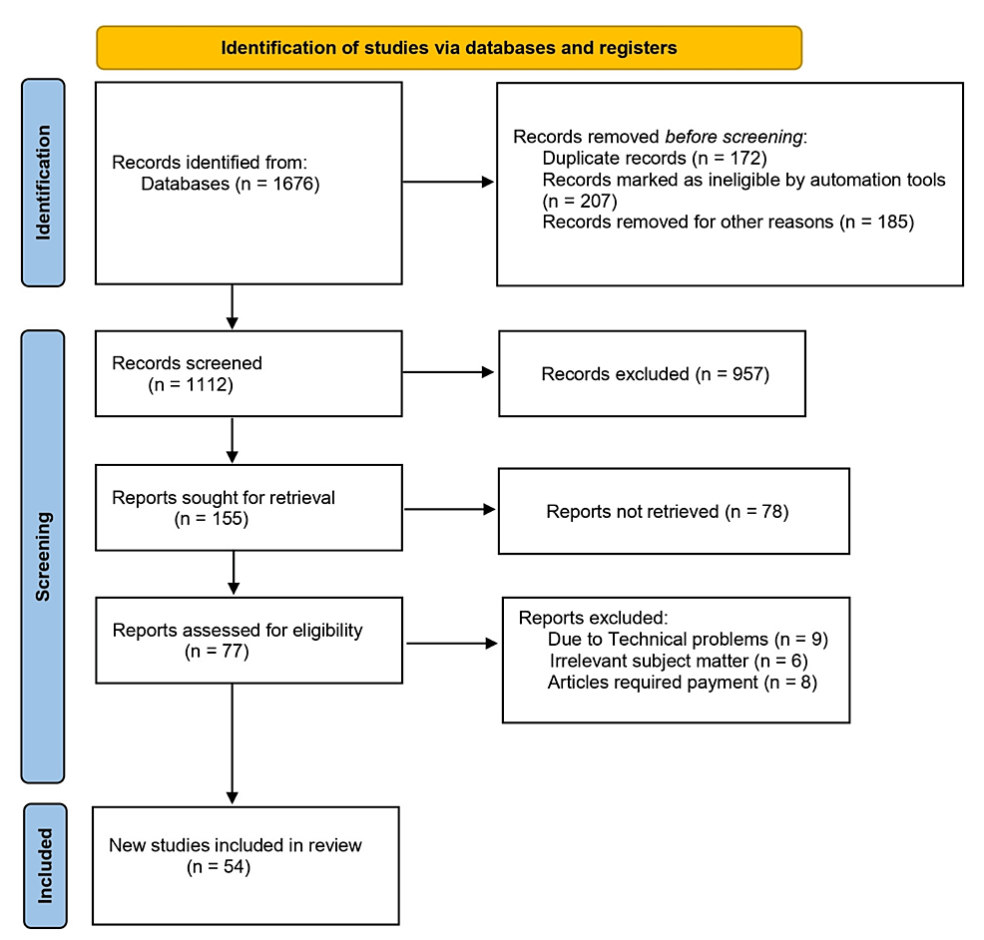
PRISMA flow diagram for literature search Adapted from the Preferred Reporting Items for Systematic Reviews and Meta-analyses (PRISMA) guidelines.

Articles, reviews, and reports were selected by predefined inclusion and exclusion criteria. Inclusion criteria required that the chosen sources be written in the English language, address recent advances in the application of AI in cancer research and clinical outcomes, explore the integration of AI in different aspects of cancer diagnosis and treatment, and encompass studies conducted between 2006 and the present year. This stringent search methodology was designed to collect an extensive and current body of literature, facilitating a thorough and up-to-date analysis of the subject matter. The data extracted from these selected studies were thoroughly analyzed and organized to provide a comprehensive understanding of the role of AI in advancing cancer research and its potential impact on clinical practice (Figure 1).

Discussion

Different AI Applications in Biomedical Cancer Research

AI has significantly benefited biomedical cancer research in several fields. AI can help in the creation of new medications and treatments, improve early cancer detection through image analysis, and personalize treatment plans based on patient information. Researchers can now analyze large datasets and spot tiny trends, thanks to ML and DL algorithms, which can aid with cancer prognosis and early diagnosis [16]. In addition, AI is essential to precision medicine since it allows doctors to customize a patient's course of therapy based on a variety of criteria, including their genetic composition. AI speeds up the process of finding promising new drugs and expedites the identification phase of the drug discovery process. Together, these applications hold out the possibility of more effective, individualized, and efficient methods for fighting cancer in the realm of biomedical research [2].

Essential components of cancer diagnosis and treatment have played a significant role in optimizing and improving these bellow aspects.

Radiodiagnosis

Modern medical imaging systems are incorporating a range of ML techniques, including those found in technologies like computed tomography (CT) and magnetic resonance imaging (MRI). CT, a radiological imaging method, can capture volumetric and morphological details of a patient's anatomical structure in medical images. A contemporary CT scanner can perform a comprehensive scan of a patient, covering everything from head to toe, with exceptional resolution [17]. Another widely used medical imaging system is MRI. MRI excels at distinguishing soft tissues, making it particularly suitable for examining the interior of joints or ligaments. However, MRI can be applied to produce images of nearly any part of the body, provided there are variations in soft tissue density to capture and it helps to identify the cancer cells [18]. Neural networks and DL algorithms are also employed in the process of brain segmentation. Together, they offer a semi-automated approach for categorizing brain MRI scans into cancerous regions and healthy tissue [19]. Thirty percent of newly diagnosed instances of cancer in women are breast cancer, making it a common disease. One often utilized technique in the diagnosis of breast cancer is ultrasonography. Enhancing breast ultrasound image segmentation into functional tissues can improve breast density measurement, tumor location, and treatment response evaluation. However, radiologists must manually segment patients, which takes time and requires a certain level of expertise. Automated segmentation of ultrasound images can assist in overcoming these obstacles. Convolutional neural network (CNN)-based segmentation has been shown in a recent study to be capable of reliably classifying four main tissues from 3D images: fatty tissue, mass, fibro-glandular tissue, and skin. Future improvements in clinical breast cancer diagnosis could benefit from this development [20].

Radiotherapy

AI is making significant strides in the realm of radiotherapy within biomedical cancer research [21]. AI technologies are proving instrumental in enhancing the precision and effectiveness of cancer treatment. In radiotherapy, AI is utilized for several crucial tasks, including the precise delineation of tumor boundaries, enabling more accurate treatment planning [10]. This ensures that radiation therapy is targeted directly at cancerous cells while sparing healthy tissues, thereby minimizing side effects. AI-driven algorithms can efficiently process vast amounts of patient data and medical images, aiding in treatment optimization [22]. Moreover, AI assists in real-time treatment monitoring, allowing for prompt adjustments to the therapy plan based on a patient's response [23]. The integration of AI in radiotherapy not only elevates treatment outcomes but also holds the potential for more personalized and efficient cancer care. This technological advancement is contributing significantly to the ongoing progress in biomedical cancer research [21].

Chemotherapy

In the realm of biomedical cancer research, the application of AI is making significant strides, particularly in the field of chemotherapy [24]. AI is playing a crucial role in refining and customizing chemotherapy treatments for cancer patients [25]. By analyzing extensive datasets, AI enables oncologists to create treatment plans tailored to each patient's unique genetic and molecular characteristics, increasing the chances of treatment success while minimizing potential side effects [16]. Also, AI-powered predictive models assist in estimating a patient's response to specific chemotherapy regimens, leading to a more personalized and targeted approach [26]. This integration of AI in chemotherapy not only improves treatment outcomes but also offers the potential for more effective and less invasive cancer care, contributing to ongoing advancements in biomedical cancer research [25].

Immunotherapy

AI encompasses the use of machines to replicate intelligent actions, addressing complex tasks with minimal human intervention. This involves concepts like ML and DL [27]. AI's integration into the field of medicine is enhancing healthcare systems across various domains, including confirming diagnoses, assessing risk, analyzing data, forecasting outcomes, monitoring treatments, and offering virtual health support [28]. These applications have the potential to revolutionize and reshape the practice of medicine. In the context of immunotherapy, AI is employed to uncover underlying immune patterns linked to responses indirectly and to directly predict responses to immunotherapy [29]. AI-driven analysis of high-throughput genetic sequences and medical imagery yields valuable insights for cancer immunotherapy management, contributing to patient selection, treatment optimization, and personalized prognosis prediction [30]. Cancer immunotherapy has emerged as a pivotal component of systematic cancer treatment, working in conjunction with traditional approaches such as chemotherapy, radiotherapy, and surgery [31]. This integration is bolstered by the advancements in biotechnologies, which have deepened our understanding of the molecular intricacies of tumors. Immunotherapy stands out as a groundbreaking approach, harnessing the patient's immune system to combat a wide range of cancers, marking a significant milestone in the treatment of this disease [32]. Through the restoration of the body's normal immune response and the resuscitation of immunological processes to regulate and eradicate malignant growths, cancer immunotherapy aims to treat tumors [33]. The release and presentation of tumor antigens, the activation of effector T cells, the migration and infiltration of T cells into tumor tissues, and the subsequent recognition and destruction of tumor cells by these activated T cells are some of the crucial steps in this complex process [34]. Many immunological markers and signatures are analyzed before treatment initiation to identify correlations with cancer patients' receptivity to immunotherapies [35].

Targeted Therapy

AI is making remarkable inroads in the realm of biomedical cancer research, particularly in the context of targeted therapy [24]. AI technologies are proving to be invaluable tools in the quest to develop more precise and effective treatment strategies for cancer patients. By analyzing complex datasets and patient profiles, it enables oncologists to tailor therapies to the individual characteristics of each patient's cancer, offering the potential for more personalized and targeted treatments [25]. This approach can enhance treatment outcomes, minimize side effects, and contribute to the ongoing progress in the field of biomedical cancer research [36]. It can assist in the identification of specific molecular targets for therapy, helping researchers and clinicians make informed decisions about which drugs or interventions are most likely to be effective for a particular patient, ushering in a new era of precision medicine in the fight against cancer [37].

Surgery

Surgical removal of tumors is frequently a critical step in cancer treatment. One subdomain of AI is utilized in computer-assisted surgery (CAS), which has become an integral part of routine clinical procedures. CAS has significantly enhanced the efficiency and effectiveness of managing oncological conditions requiring surgical intervention, making a substantial impact on the field of cancer care [38]. Computer vision (CV) is extensively utilized in image-guided navigation, referring to a system created to aid surgeons by using preoperative CT images for guidance [39]. This technology facilitates the exploration of a patient's anatomy, the identification of both pathological and vital structures, and the formulation of surgical plans for their removal or preservation. It combines radiological imaging with precise tracking technologies integrated into surgical instruments, aligning them with the patient's coordinate system. The system can detect and highlight structures of interest, even those that are not readily visible, greatly aiding surgeons in safely and effectively navigating toward their surgical targets [38]. Currently, image guidance and navigation systems have seen significant utilization, especially in the fields of neurosurgery and orthopedic surgery. They are particularly valuable in surgeries where the anatomical structures are not significantly altered by tissue displacement or organ movement [40].

In situations where surgical procedures involve significant alterations in anatomical structures due to tissue manipulation, computer-based navigation systems have seen limited application. However, substantial efforts have been dedicated to introducing AI-driven surgical navigation techniques in surgeries where dissecting planes can cause significant changes in anatomy, as is the case in abdominal surgery. Consequently, these innovative techniques in research can provide valuable insights and guidance for revealing hidden anatomical features. For instance, they can help in visualizing the precise positioning of vital structures like the aorta and the ureter concerning surgical instruments in laparoscopic rectal surgery [41]. Another noteworthy achievement is the creation of computer-assisted liver maps for use in liver cancer surgery [42]. In the future, there is a growing potential for mapping additional structures on CT images, making them accessible for image-guided abdominal surgery. This expansion may encompass structures such as the spleen, pancreas, and esophagus [43]. Robotic prostatectomy has the potential to emerge as the leading surgical approach for treating prostate cancer [44]. The field of robotic surgery holds great promise, with high expectations. Shortly, we anticipate the integration of assistive systems with surgical robots. These incorporated CV technologies will play a pivotal role by offering surgeons insights into anatomical structures and resection margins. This will be achieved through real-time comparisons of intra-operative data with a vast database of millions of reference images [45].

Nanotechnology

AI with the advancement of nanotechnology-based products has the potential to revolutionize health care, especially in the realm of cancer management. AI can efficiently handle and analyze large datasets, allowing for the creation of tailor-made precision nanomedicines [46]. By leveraging AI, molecular profiling and early patient diagnosis can become more accurate. Additionally, AI can optimize the development process of nanomedicines by fine-tuning their properties, achieving effective drug synergy, and minimizing potential nontoxicity [47]. This innovation ultimately enhances the precision of treatment, personalized dosing, and the overall efficacy of nanomedicines [48]. Current cancer treatments come with their share of side effects, and the atypical extracellular matrix (ECM) in solid tumors poses a challenge by impeding the penetration of therapeutic agents and immune cells. In response, recent endeavors have focused on the development of anticancer nanomedicines to overcome these constraints [49]. Nanomedicines represent a fusion of nanotechnology and medicine with applications in disease diagnosis, monitoring, and treatment. They can be directly administered to patients in clinical settings or integrated into medical bio-devices, nano-biosensors, and biological machines to enhance healthcare capabilities [50]. A variety of nanomedical products have made their way into the global market, with applications in the treatment of several cancer types, including ovarian cancer, breast cancer, lung cancer, pancreatic cancer, acute myeloid leukemia, and more. To maximize the effectiveness and safety of nanomedicines, it is crucial to consider the complex interactions between these nanocarriers and tumor cells. This includes processes like internalization, responses within the tumor microenvironment, intracellular distribution, and the therapeutic actions they induce. While the integration of nanomedicines and AI is still in its early stages, AI can play a pivotal role in these processes [51]. For example, AI can predict interactions between nanocarriers and their encapsulated drugs, biological mediators, or cell membranes. It can also estimate drug encapsulation efficiency and model drug release kinetics from nanocarriers using advanced algorithms. These AI-driven approaches can significantly optimize nanopharmaceutical formulations, enhancing the transport and targeting capabilities of nanomedicines [52].

A careful analysis of the advantages and disadvantages of AI approaches in cancer research is necessary for a thorough evaluation of these methods. AI can greatly improve cancer diagnosis, prognosis, and medication discovery speed and accuracy [6]. These technologies do have certain drawbacks, such as the requirement for sizable, excellent datasets and the possibility of biases in the data. Furthermore, it may be difficult to comprehend the reasoning behind some judgments made, which raises questions about the interpretability of AI-driven results [53]. We found that the AI system is able to provide patients with triage and diagnostic information with a level of clinical accuracy and safety comparable to that of human doctors. Numerous studies have focused on clinical decision support systems that utilize AI to aid oncologists in making treatment decisions. These systems are often designed to provide evidence-based recommendations and assist doctors in selecting the most suitable treatment options based on patient-specific data. AI systems assist in determining the optimal treatment for prostate cancer patients. The AI system's recommendations were compared to those of expert oncologists. The study found that the AI system's recommendations aligned with those of the experts in most cases and, in some instances, suggested alternative treatment options [54]. Emerging trends in the subject can be seen by contrasting and comparing different studies, such as the growing importance of AI in personalized and precision medicine. This in turn highlights the current gaps in the literature, including the need for more clinical validation and ethical considerations. AI has significant implications for the advancement of cancer research, with the potential to result in better patient outcomes, early identification, and more effective treatments [16]. To fully utilize AI for cancer patients and the oncology community at large, it is imperative to address these constraints and research gaps. A summary of all articles included in this review is listed in Table 1.

**Table 1:** Summary table of studies included in the review.

Authors	Year	Country	Findings
Vamathevan J et al. [2]	2019	United Kingdom	Artificial intelligence (AI) technologies have made significant advancements in various clinical fields, such as ophthalmology, radiology, and dermatology, achieving performance levels comparable to human experts and impacting a wide range of clinical indications, including basic biology and pharmacology.
Alharbi F et al. [3]	2023	USA	Machine Learning (ML) offers a diverse set of algorithms that enhance accuracy in decision-making and predictions when supplied with substantial, high-quality data.
Krizhevsky A et al. [4]	2012	Canada	Deep learning (DL) employs neural network models to address challenges that traditional machine learning (ML) methods may be unable to tackle.
Iqbal MJ et al. [5]	2021	Pakistan	Collaborative AI systems enable real-time knowledge sharing among researchers, holding the potential to benefit millions of people by advancing healthcare and medical research.
Sebastian AM et al. [6]	2022	India	DL and ML models on distributed datasets provide reliable, fast, and effective solutions for cancer therapy.
Ahmad Z et al. [7]	2021	Afghanistan	The future of personalized medicine in cancer diagnosis and treatment relies on AI and ML in clinical applications for quicker and tailored treatment solutions.
Bhinder B et al. [8]	2021	USA	Advanced federated learning models enhance distributed data analysis. Cutting-edge tools like virtual biopsies, natural language processing for health trajectory inference, and whole-blood cancer diagnosis through deep sequencing hold promise for clinically significant advancements.
Senthil Kumar K et al. [9]	2023	Singapore	Diagnostic systems have evolved from basic light microscopy to advanced testing, including gene expression assays and next-gene sequencing of genomes.
Liao J et al. [10]	2022	China	In radiotherapy, AI plays a vital role in precise tumor boundary delineation, improving treatment planning accuracy.
Zamberlan F [11]	2023	USA	The success of AI interventions in cancer research relies on the availability of appropriate data for ML and DL model development.
Shao D et al. [12]	2022	China	Over the past two decades, there has been a significant surge in digital data, reigniting the potential of AI and demonstrating promise across all aspects of cancer research.
Tozzi AE et al. [13]	2022	Italy	The development of AI applications for cancer clinical research has been a prominent subject in public discourse about AI.
Bohr A et al. [14]	2020	Denmark	Digital pathology, radiology, and genomic data are employed to optimize combination regimen design and determine precise chemotherapy and immunotherapy dosing.
Strickland E et al. [15]	2019	Turkey	IBM has been a pioneer in implementing AI in clinical settings, with a dedicated focus on this endeavor.
Johnson KB et al. [16]	2021	USA	AI advances cancer research with new treatments, early detection, and personalized care.
Salehjahromi M et al. [17]	2018	USA	CT, a radiological imaging technique, captures detailed volumetric and anatomical information in medical images.
Siddique S et al. [18]	2020	Canada	MRI can generate images of various body parts, relying on soft tissue density variations, aiding in cancer cell identification.
Zhang Z et al. [19]	2019	USA	Neural networks and deep learning algorithms play a role in brain segmentation.
Xu Y et al. [20]	2019	China	Improved breast ultrasound image segmentation benefits breast density measurement, tumor localization, and treatment response assessment.
Wahid KA et al. [21]	2022	USA	AI is advancing radiotherapy in biomedical cancer research.
Grégoire V et al. [22]	2020	Germany	AI algorithms efficiently handle extensive patient data and medical images, enhancing treatment optimization.
Bajwa J et al. [23]	2021	United Kingdom	AI enables real-time treatment monitoring, facilitating prompt therapy plan adjustments based on patient responses.
Kashyap BK et al. [24]	2023	India	AI is making notable advancements in biomedical cancer research, especially in the domain of chemotherapy.
Farina E et al. [25]	2021	Brazil	AI is pivotal in tailoring and refining chemotherapy treatments for cancer patients.
Russo V et al. [26]	2022	Italy	AI predictive models aid in anticipating a patient's response to specific chemotherapy regimens, promoting personalized and targeted treatment strategies.
Yang Y et al. [27]	2022	China	AI involves machine-based replication of intelligent actions, handling complex tasks with minimal human intervention, including machine learning and deep learning.
Ahuja AS et al. [28]	2019	USA	The integration of AI into medicine is elevating healthcare across domains, encompassing diagnosis confirmation, risk assessment, data analysis, outcome prediction, treatment monitoring, and virtual health support.
Xu Z et al. [29]	2021	China	Some applications have the potential to revolutionize and reshape the practice of medicine.
Chaudhary N et al. [30]	2021	India	AI-driven analysis of genetic sequences and medical imagery offers valuable insights for cancer immunotherapy, benefiting patient selection, treatment optimization, and personalized prognosis prediction.
Thompson RF et al. [31]	2018	USA	Cancer immunotherapy has become a key component of comprehensive cancer treatment, complementing traditional methods like chemotherapy, radiotherapy, and surgery.
Hanahan D et al. [32]	2012	Switzerland	Advancements in biotechnologies have deepened our understanding of tumor molecular complexities, reinforcing the integration of immunotherapy with traditional cancer treatments.
Waldman AD et al. [33]	2020	USA	Cancer immunotherapy seeks to treat tumors by restoring the body's immune response and reviving immunological processes to control and eliminate malignant growths.
Kim SK et al. [34]	2022	Korea	Key steps in this complex process include the release and presentation of tumor antigens, activation of effector T cells, T cell migration into tumor tissues, and the subsequent recognition and destruction of tumor cells by activated T cells.
Zhang Y et al. [35]	2020	China	Numerous immunological markers and signatures are examined prior to treatment commencement to identify associations with cancer patients' responsiveness to immunotherapies.
Bustin SA et al. [37]	2023	United Kingdom	It aids in pinpointing specific molecular therapy targets, enabling informed decisions on effective drugs or interventions for individual patients, and ushering in a new era of precision medicine in the battle against cancer.
Deo S et al. [38]	2011	India	Computer-assisted surgery (CAS) has notably improved the efficiency and effectiveness of managing surgical oncological cases, exerting a substantial impact on the field of cancer care.
Cabral BP et al. [39]	2023	Brazil	Computer vision (CV) is widely employed in image-guided navigation, assisting surgeons through the use of preoperative CT images for guidance.
Mezger U et al. [40]	2013	Germany	Surgeries in which anatomical structures are minimally affected by tissue displacement or organ movement.
Okada T et al. [41]	2020	Japan	Significant efforts have been focused on implementing AI-driven surgical navigation in procedures where dissecting planes can lead to substantial anatomical changes, as seen in abdominal surgery.
Kingham TP et al. [42]	2012	USA	A noteworthy achievement is the development of computer-assisted liver maps for application in liver cancer surgery.
Nickel F et al. [43]	2013	Germany	In the future, there is increasing potential to map additional structures on CT images, expanding their utility in image-guided abdominal surgery.
Sood A et al. [44]	2014	USA	Robotic prostatectomy has the potential to become the primary surgical approach for prostate cancer treatment.
Ngiam KY et al. [45]	2019	Singapore	The field of robotic surgery is filled with great promise and high expectations.
Yoon HY et al. [46]	2018	Korea	AI, coupled with advancements in nanotechnology, has the potential to revolutionize healthcare, particularly in cancer management. AI's capacity to efficiently process and analyze large datasets enables the development of customized precision nanomedicines.
Li Z et al. [47]	2022	China	AI can optimize the development of nanomedicines by fine-tuning properties, enhancing drug synergy, and minimizing potential non-toxicity.
Green MR et al. [48]	2006	USA	Innovation ultimately improves treatment precision, personalized dosing, and the overall effectiveness of nanomedicines.
Klein K et al. [49]	2019	Netherlands	Recent efforts have been directed toward creating anticancer nanomedicines to overcome these limitations.
Prasad M et al. [50]	2017	India	Nanomedicines combine nanotechnology and medicine, finding applications in disease diagnosis, monitoring, and treatment.
Edis Z et al. [51]	2021	United Arab Emirates	The fusion of nanomedicines and AI is in its early stages, AI can play a crucial role in these processes.
Adir O et al. [52]	2020	Israel	AI can forecast interactions between nanocarriers and their enclosed drugs, biological agents, or cell membranes.
Amann J et al. [53]	2020	Switzerland	Technologies do come with certain limitations, such as the need for large, high-quality datasets and the potential for data biases.
Alexander-Bryant AA et al. [36]	2013	USA	The approach can improve treatment outcomes, reduce side effects, and contribute to ongoing advancements in the field of biomedical cancer research.
Levine AB et al. [1]	2019	Canada	Artificial intelligence (AI) has found extensive use in various medical specialties.
Baker A et al. [54]	2020	United Kingdom	AI systems can offer patients clinical triage and diagnostic information as accurately and safely as human doctors.

## Conclusions

In conclusion, AI has shown to be a potent tool in the fight against cancer. Better patient outcomes could result from its use in research, diagnosis, and therapy. AI applications in biomedical cancer research offer significant potential, but they also come with several challenges such as validation and clinical adoption, bias and fairness, data privacy and security, data quality and quantity, and interpretability. To fully realize AI's promise in enhancing cancer clinical research, we must continue to be alert in resolving issues with data quality, interpretability, and ethical issues. The future is bright, and we can anticipate a more individualized and successful approach to controlling and treating cancer as we continue to hone and broaden AI-driven solutions.
